# Investigating the Associations of Self-Rated Health: Heart Rate Variability Is More Strongly Associated than Inflammatory and Other Frequently Used Biomarkers in a Cross Sectional Occupational Sample

**DOI:** 10.1371/journal.pone.0117196

**Published:** 2015-02-18

**Authors:** Marc N. Jarczok, Marcus E. Kleber, Julian Koenig, Adrian Loerbroks, Raphael M. Herr, Kristina Hoffmann, Joachim E. Fischer, Yael Benyamini, Julian F. Thayer

**Affiliations:** 1 Mannheim Institute of Public Health, Social and Preventive Medicine, Mannheim Medical Faculty, Heidelberg University, Mannheim, Germany; 2 The Ohio State University, Department of Psychology, Columbus, Ohio, United States of America; 3 Institute of Occupational and Social Medicine, Medical Faculty, Heinrich-Heine-University Düsseldorf, Düsseldorf, Germany; 4 Department of Clinical Psychology, University of Amsterdam, Amsterdam, The Netherlands; 5 Bob Shapell School of Social Work, Tel Aviv University, Tel Aviv, Israel; University of Utah, UNITED STATES

## Abstract

The present study aimed to investigate the possible mechanisms linking a single–item measure of global self-rated health (SRH) with morbidity by comparing the association strengths between SRH with markers of autonomic nervous system (ANS) function, inflammation, blood glucose and blood lipids. Cross–sectional comprehensive health–check data of 3947 working adults (age 42±11) was used to calculate logistic regressions, partial correlations and compare correlation strength using Olkins *Z*. Adjusted logistic regression models showed a negative association between SRH (higher values indicating worse health) and measures of heart rate variability (HRV). Glycemic markers were positively associated with poor SRH. No adjusted association was found with inflammatory markers, BP or lipids. In both unadjusted and adjusted linear models Pearson’s correlation strength was significantly higher between SRH with HRV measures compared to SRH with other biomarkers. This is the first study investigating the association of ANS function and SRH. We showed that a global measure of SRH is associated with HRV, and that all measures of ANS function were significantly more strongly associated with SRH than any other biomarker. The current study supports the hypothesis that the extent of brain–body communication, as indexed by HRV, is associated with self-rated health.

## Introduction

Self-rated health (SRH), a simple question asking individuals to rate their health in general, has consistently been found to predict mortality, morbidity, and other health outcomes [1]. This is not surprising in itself, as SRH is associated with many indicators of physical and mental health (see meta-analysis by Pinquart [2]), such as functional ability, depression, and chronic pain, as well as with social risk factors such as socioeconomic status (e.g. income disparities), demographics (age and gender), work stress and leadership [3]. The more intriguing finding is that in most studies that have examined this, SRH remained a significant predictor of future health outcomes even after controlling for many relevant covariates that include known risk factors for poor health [1].

The single item SRH measure has demonstrated good reproducibility, reliability, and strong concurrent and discriminant scale performance with an established more detailed subjective health status measure [4]. Its validity has been consistently supported by hundreds of studies reporting its associations with physician ratings and other health measures and its prediction of future mortality and morbidity. Further support for its role as an integrative evaluation of one’s health and not a momentary assessment comes from studies showing that it is not sensitive to mood inductions [5] or recent acute illnesses [6], and, while it has been found to be associated with measures of personality, its associations with objective medical burden remain even after controlling for personality [7]. Furthermore, a 22-year follow up of the General Social Survey showed an increasing predictive validity of self-rated health on of mortality [8].

One of the explanations proposed for this independent association of SRH with mortality and morbidity is that SRH is more inclusive than the covariates typically used in studies and as a single item, provides an optimal integration of many types of information, including external (e.g. diagnoses) and internal information (e.g. bodily sensations) [9,10]. Thus, SRH may reflect diseases at pre-clinical stages as well as more accurately reflect the cumulative effects of comorbidity [9,11]. This explanation is supported by findings showing that SRH is strongly associated with tiredness [12], declines in timed gait [13] and walking speed [14], and that vigor interacts with functional capacity to predict changes in SRH [15]. Moreover, the "valid core" of SRH has been found to include feelings of fatigue, lack of energy, and diminished activity [6].

In line with these self-reports, SRH has been found to be associated with a wide range of biomarkers [16,17], including inflammatory markers [18], even after controlling for a variety of health and psychosocial measures [19]. The association of SRH with mortality is similar to [20] or even superior to that of a panel of biomarkers [21,22]. In sum, while the association of SRH with future health diminishes with increasing adjustments, it is typically not eliminated, suggesting that SRH is a predictor of mortality because SRH reflects the state of the human organism and is likely to be based at least in part on interoceptive processes [11].

A possible but as yet uninvestigated mechanism that could link the various sources of information that feed into SRH is autonomic nervous system (ANS) activity. One widely used measure of ANS activity is the beat-to-beat variations in the cardiac rhythm as indexed by heart rate variability (HRV). HRV may be more than just an index of healthy heart function and may in fact serve as an easily measured output of the brain’s integrative system for adaptive regulation [23]. Like SRH, HRV has been independently associated with morbidity and mortality from a wide range of disorders including metabolic syndrome (MetS) [24], low-grade inflammation [25,26], fatigue [27], work stress [28], and cardiovascular disease [29]. Thus, HRV, like SRH, may be a more inclusive index of health and may be a more integrative measure of external and internal health-related information. In sum, HRV seems to be a promising physiological correlate of SRH yet surprisingly, based on a systematic literature search in Medline (via PubMed) and PsychInfo (1^st^ April 2014), the relationship between SRH and HRV has not been investigated.

Therefore, the following analysis aimed to investigate (1) the associations between a single–item measure of global SRH and HRV in a large sample of working adults; and (2) the relative strength of associations between global SRH and HRV versus other frequently used biomarkers including inflammatory markers. *It is hypothesized that SRH would be associated with ANS activity as indexed by HRV and that this association would be more pronounced in comparison to other frequently used but less inclusive biomarkers*.

## Methods

### Study Population and Design

A total of 9730 workers employed in four distinct plants were invited between September 2009 and May 2011 to a voluntary comprehensive health–check comprising a questionnaire and a medical examination in Southern Germany. A total of 4881 employees (mean age 41 SD 11 from 18–65 years, 22% females) accepted the invitation to the health-check (response rate 50.2%). Complete information on all used measurements was necessary to rule out composition effects when comparing coefficients between measurements resulting in an analysis sample of N = 3947 (80.9%). All participants were Caucasian and assessed by an agent independent from the employer who conducted the health risk assessments and data collection (HealthVision Ltd., Berlingen, Switzerland). The Ethical Committee of the Mannheim Medical Faculty, Heidelberg University approved this secondary data analysis (2010-296E-MA). All participants gave written informed consent prior to examination. For more details on measurements and population please see [24,30].

### Measures

SRH was assessed using a single item asking about overall health status based on the past four weeks: “In general, would you say your health is: Excellent (1), Very Good (2), Good (3), Fair (4), Poor (5)”.

### Biomarkers

Raw beat–to–beat intervals (IBI) from 24h HR-recordings were analyzed by researchers at the Centre for Neuropsychological Research (University of Trier, Germany) according to the Task Force Guidelines [31]. The 24-hour IBI-data were decomposed into 5.35 minutes blocks. The “ANS-Explorer” Software [32] was used to calculate time domain and frequency domain (Fast Fourier Transform-based) parameters as well as artifact rate per block. The mean IBI, root mean square of successive differences (RMSSD), standard deviation of all N–N (normal–to–normal) intervals (SDNN), frequency domain power in the low (LF) (0.04–0.15 Hz) and high frequency (HF) (0.15–0.4 Hz) were categorized into tertiles (Table 1). RMSSD and HF are known to reflect primarily parasympathetic mediated HRV while SDNN and LF are known to reflect both, sympathetic and parasympathetic mediated HRV.

**Table 1:** Classification of biomarkers into (clinical) categories.

			Categories or Quantiles			
	Variable	Condition	1	2	3	4
HR & HRV	BPM		<71	71–78	>78	
	IBI (ms)		>872	786–872	<786	
	RMSSD (ms)		>31	22–31	<22	
	SDNN (ms)		>67	55–67	<55	
	LF (ms^2^)		>879	557–879	<557	
	HF (ms^2^)		>263	126–263	<126	
Glycemic Status	FPG (mg/dl)		<80	80–<100	100–<126	≥126
	HbA_1c_ (%)		4–<6	6–<6.5	≥6.5	
Inflammation Status	CRP (mg/L)		<5	≥5		
	WBC (μL)		<5.5	5.5–6.6	>6.6	
Lipid Status	LDL (mg/dl)		<160	≥160		
	HDL (mg/dl)	females	≥55	35–<55	<35	
	HDL (mg/dl)	males	≥65	45–<65	<45	
	CHOL (mg/dl)	age <20	<170	≥170		
	CHOL (mg/dl)	age 20–<29	<200	≥200		
	CHOL (mg/dl)	age 30–<39	<220	≥220		
	CHOL (mg/dl)	age 40+	<240	≥240		
	TRIG (mg/dl)		<200	≥200		
Blood Pressure	Sys BP (mmHg)	In combination with Dia BP	<120 &	120–139 &	≥140 &	≥140 &
	Dia BP (mmHg)	In combination with Sys BP	<90	<90	<90	≥90
	MAP (mmHg)		<92.7	92.7–101.8	>101.8	

CRP = C-reactive protein. WBC = White blood count. FPG = Fasting plasma glucose. HbA_1c_ = glycosylated hemoglobin. LDL = Low density lipoprotein. HDL = High density lipoproteins. CHOL = Cholesterol. TRIG = Triglyceride. Sys BP = Systolic blood pressure. Dia BP = Diastolic BP. MAP = Mean arterial pressure. IBI = Inter-beat interval. RMSSD = Square root of the mean of successive differences. LF = Low frequency power. HF = High frequency power. SDNN = Standard deviation of normal-to-normal intervals.

Demographic, medical and lifestyle variables were obtained from an online questionnaire. The questionnaire had to be completed prior to being able to schedule the medical examination. All participants were enrolled and examined between 10 a.m. and 5 p.m. on a typical workday (Monday to Friday) during work hours. Upon arrival a medical examination was performed and the HR-recorder was attached. The next morning, between 7 a.m. and 9 a.m., a fasting blood sample was collected from all individuals. Samples were transported to a commercial laboratory (Synlab, Augsburg, Germany) within 2 hours of sample collection and analyzed within 24-hours.

Inflammatory markers (C-reactive protein [CRP], white blood count [WBC]), blood glucose (Fasting plasma glucose [FPG], glycosylated hemoglobin [HbA_1c_]), and blood lipids (Total cholesterol [CHOL], triglyceride [TRI], low density lipoprotein [LDL], high density lipoprotein [HDL]) were determined using routine laboratory analyzers (for details see [24]) Blood pressure (BP) using the oscillometric technique was recorded twice using the CRITIKON Dinamap Portable Adult/Pediatric and Neonatal Vital Signs Monitor (Model 8100). Measurements were taken from the dominant arm in the seated position after a 5-minute rest period. A study physician repeated the reading using sphygmomanometry if one or more BP values exceeded 135mmHg (systolic) or 90mmHg (diastolic). The arithmetic mean of all two to three measurements was calculated.

### Other covariates

Work stress was assessed by a validated short version of the Effort–Reward Imbalance (ERI) questionnaire [33]. According to a predefined algorithm, a ratio between the “effort” and “reward” scales was calculated to quantify the degree of mismatch between high “cost” and low “gain” at the individual level (weighted by number of items). Here, values >1 indicate failed reciprocity between efforts spent at work and rewards received in turn.

Subjective sleep quality was assessed by a continuous score based on a translation of the 4–item Jenkins sleep quality scales with higher values indicating worse sleep quality [34]. Socio-economic measures were span of control (0, 1–4, 5–20, >20) and hierarchical position (division manager, team manager, foreman, skilled worker, unskilled worker). Behavioral factors included A) smoking status (non-smokers, ex-smokers, or current smokers), B) alcohol drinking during the past 6 months by four questions on the frequency of wine, beer, spirits or cocktails (6 to 7 days a week; 3 to 5 days a week; Once or twice a week; Once or twice a month; Never) and C) frequency of vigorous physical activity during the past 6 months by one question (three times or more often per week; More than once a week; About once a week; One to three times per month; Hardly ever).

The components of the MetS were defined according to the joint interim statement to harmonize the MetS [35]. We considered MetS to be present if at least three of the following criteria were met: triglycerides (TRI) ≥1.7 mmol/l (150 mg/dl) or use of medication for hypertriglyceridemia was reported; high-density lipoprotein (HDL) ≤1.0 mmol/l (40 mg/dl) for males and ≤1.3 mmol/l (50 mg/dl) for females, waist circumference ≥94 cm for males or ≥80 cm for females, systolic BP ≥130 mmHg and/or diastolic BP ≥85mmHg or use of an anti-hypertensive medication was reported, fasting plasma glucose (FPG) ≥5.6mmol/l (100mg/dl) or use of a hypoglycemic medication was reported.

### Statistical Analysis

The distribution of all continuous variables was determined and transformations were used as necessary to meet the assumptions of modeling. Differences between the full sample and the analysis sample were determined by t-test for continuous and χ^2^ test for categorical variables. A four-stage approach was applied:

First, bivariate associations between SRH and continuous biomarkers were assessed using Bonferroni-corrected Pearson’s correlation as well as nonparametric Kendall’s rank correlation coefficient τ_b_. Compared to Pearson’s correlation, Kendall τ_b_ does neither assume a normal distribution nor equidistant scaling of the correlated variables. In addition, Kendall τ_b_ makes adjustments for ties. Values of τ_b_ range from −1 (100% negative association) to +1 (100% positive association). A value of zero indicates the absence of association. This could be important, since the various biomarkers are usually neither normally distributed nor equidistant to a five-point Likert scale (SRH). On the other hand, Pearson correlations are easier to compare.

Second, multiply–adjusted partial correlation coefficients and partial rank correlation coefficients Kendall τ_b_ were used to reflect the association between SRH and a biomarker independent of covariates (demographics, lifestyle, sleep quality, and work stress) in the model.

Third, we used one sample Olkin’s *Z* to systematically compare the correlation strength (gained from step one and two) between the associations of SRH and each biomarker additionally adjusting for multiple comparisons by Bonferroni-correction. The null hypothesis of Olkins *Z* is that the correlation strength between SRH and biomarker A is equal to the correlation strength of SRH and biomarker B.

Fourth, biomarkers were categorized into either clinically relevant categories or into quantiles if no clinical cut offs exist (Table 1) to show the relative strength of association (both unadjusted and adjusted) in odds ratios by each category for all biomarkers, which are then summarized in a forest plot. Here, the point estimate and it’s respective confidence interval can be easily compared across the logistic regressions per biomarker. SRH was dichotomized with individuals indicated fair (12.79%) or poor (0.91%) SRH versus those indicating good (49.91%), very good (31.42%) or excellent (4.97%) SRH. Hierarchical multiple logistic regression models with dichotomized SRH as the dependent and each categorized biomarker as the independent variable adjusting block wise for demographics, lifestyle, sleep quality, and work stress were calculated. Likelihood–ratio tests were applied after each block to test logistic model improvement. The overall contribution of variables to the model was tested using Wald–tests. Covariates that had no association with SRH and did not contribute significantly to the overall model fit in logistic regressions were excluded from final analysis.

Imputing missing SRH values (2.01%) by missing value regression had no impact on any results and are reported here. A total of 3947 individual data sets with complete data regarding questionnaire, medical data, and HRV assessment remained. STATA 12.1MP (College Station, TX) was used for data management and analysis.

## Results

The study sample consisted primarily of male workers (78%). The majority of participants were skilled workers (64%) as well as project or division managers (29%) without (78%) and with (22%) leadership responsibilities. Healthy behavior seems to be prominent with 81% non-smoker, 62% drinking alcohol maximal twice a week, 70% doing sweat–rich activities at least once a week, and having less than three components of the MetS (77%). We found some differences between the full sample and the analysis sample regarding blood lipids and glucose levels but the magnitudes of the differences are below clinically relevant levels (e.g. FPG-difference = 2.08mg/dl) (Table 2).

**Table 2:** Differences between analysis sample and missing sample.

		Analysis Sample (N = 3947)		Missing Sample			Differences
	Variable	mean	sd	mean	sd	N	delta^$^
	SRH	2.73	0.78	2.74	0.8	916	−0.01
HR & HRV	BPM#	74.62	9.24	76.49	9.5	350	**−1.87**
	IBI	838.5	107.0	816.4	104.8	350	**22.1**
	RMSSD# (ms)	30.03	13.11	26.83	12.4	350	**3.2**
	SDNN# (ms)	62.90	15.86	58.72	16.44	350	**4.18**
	LF# (ms^2^)	812.1	439.3	710.2	391.2	350	**101.9**
	HF# (ms^2^)	289.6	297.2	235	252.2	350	**54.6**
Glycemic Status	FPG (mg/dl)	87.94	11.94	90.02	16.36	585	**−2.08**
	HbA_1c_ (%)	5.62	0.38	5.69	0.5	588	**−0.08**
Inflammation Status	CRP# (mg/L)	1.70	3.78	1.66	2.9	661	0.04
	WBC (μL)	6.43	1.67	6.39	1.6	588	0.04
Lipid Status	LDL (mg/dl)	126.0	32.3	124.1	32.7	653	1.9
	HDL# (mg/dl)	57.18	14.4	54.07	14.16	672	**3.11**
	CHOL (mg/dl)	214.1	42.53	210.4	42.68	672	3.7
	TRIG (mg/dl)	129.0	84.4	136.1	93.43	672	−7.1
Blood Pressure	Sys BP (mmHg)	136.0	14.02	136.4	13.6	725	−0.2
	Dia BP (mmHg)	78.29	11.42	80.3	11.83	725	−2.01
	MAP# (mmHg)	97.6	11.47	99	11.34	725	−1.4
Covariates	Age (years)	40.91	11.48	41.94	11.54	914	−1.03
	Sleep Quality (Jenkins)	5.2	3.95	5.05	3.8	916	0.15
	No. MetS Components	1.56	1.24	1.76	1.27	666	**−0.21**
	Work Stress (ERI)	1.19	0.47	1.2	0.49	916	−0.01
	Alcohol*	2.42	1.31	2.4	1.42	798	−0.02
	Sport†	2.25	1.26	2.3	1.37	747	−0.05

$ Difference determined by T-Test (categorical variables) or χ^2^ test (metric variables).

**Bold delta values** are significant at a Bonferroni corrected 2 sided α≤0.05/24 = p≤0.002.

#log transformed to determine differences.

*Alcohol = No alcohol, 1–2 times/month, 1–2 times/week, 3–5 days/week, 6–7 days/week.

† **Sport** = Sweat rich activities 3 or more times/week, 2 times/week, 1/week, 1–3 times/month, less than 1/month.

MetS = Metabolic Syndrome. CRP = C-reactive protein. WBC = White blood count. FPG = Fasting plasma glucose. HbA_1c_ = glycosylated hemoglobin. LDL = Low density lipoprotein. HDL = High-density lipoproteins. CHOL = Cholesterol. TRIG = Triglyceride. Sys BP = Systolic blood pressure. Dia BP = Diastolic BP. MAP = Mean arterial pressure. IBI = Inter-beat interval. RMSSD = Square root of the mean of successive differences. LF = Low frequency power. HF = High frequency power. SDNN = Standard deviation of normal-to-normal intervals.

### Bivariate associations

Bonferroni–corrected correlations (both Pearson’s r and Kendall τ_b_) indicate the highest significant associations between our indices of ANS function and SRH. Higher SRH values (= worse health) were significantly and negatively associated with vagally–mediated HRV (RMSSD r = −0.22; HF r = −0.21) as well as with mixed sympathetic and parasympathetic measures (SDNN r = −0.22; LF r = −0.20). Additionally, rank order Kendall τ_b_ correlations reveal a comparable order of association strength (Table 3).

**Table 3:** Pearson’s correlation and Kendall τ_b_ of self-rated health (SRH) with ANS function measure and other biomarkers.

		Kendall τ_b_																	
**Pearson’s Correlation**	**BIOMARKER**	**SRH**	**(A)**	**(B)**	**(C)**	**(D)**	**(E)**	**(F)**	**(G)**	**(H)**	**(I)**	**(J)**	**(K)**	**(L)**	**(M)**	**(N)**	**(O)**	**(P)**	**(Q)**
	**SRH**	1	0.108*	0.048*	0.099*	0.097*	0.088*	−0.045*	0.081*	0.106*	0.044*	0.089*	0.078*	0.050*	−0.061*	−0.169*	−0.153*	−0.161*	−0.142*
	**CRP# (mg/L) (A)**	0.129*	1	0.201*	0.070*	0.100*	0.076*	−0.088*	0.068*	0.153*	0.085*	0.085*	0.091*	0.142*	−0.152*	−0.131*	−0.152*	−0.100*	−0.151*
	**WBC (μL) (B)**	0.066*	0.313*	1	0.045*	0.076*	0.029*	−0.124*	0.009	0.121*	0.044*	0.025*	0.033*	0.137*	−0.142*	−0.076*	−0.090*	−0.052*	−0.108*
	**FPG (mg/dl) (C)**	0.131*	0.110*	0.083*	1	0.220*	0.145*	−0.095*	0.130*	0.134*	0.164*	0.187*	0.192*	0.030*	−0.041*	−0.200*	−0.161*	−0.201*	−0.146*
	**HbA_1c_ (%) (D)**	0.130*	0.152*	0.130*	0.593*	1	0.157*	−0.046*	0.134*	0.090*	0.075*	0.098*	0.096*	0.029*	−0.034*	−0.152*	−0.137*	−0.149*	−0.112*
	**LDL (mg/dl) (E)**	0.115*	0.102*	0.041*	0.150*	0.171*	1	−0.071*	0.763*	0.309*	0.146*	0.206*	0.197*	−0.000	−0.008	−0.188*	−0.132*	−0.193*	−0.126*
	**HDL# (mg/dl) (F)**	−0.054*	−0.117*	−0.167*	−0.136*	−0.078*	−0.090*	1	0.091*	−0.304*	−0.125*	−0.125*	−0.133*	−0.019	0.022*	0.041*	−0.022*	0.038*	0.011
	**CHOL (mg/dl) (G)**	0.108*	0.091*	0.013	0.141*	0.151*	0.923*	0.152*	1	0.320*	0.131*	0.190*	0.181*	0.005	−0.014	−0.196*	−0.170*	−0.201*	−0.149*
	**TRIG (mg/dl) (H)**	0.1073*	0.168*	0.143*	0.209*	0.143*	0.314*	−0.412*	0.386*	1	0.190*	0.220*	0.225*	0.046*	−0.056*	−0.168*	−0.135*	−0.161*	−0.137*
	**Sys BP (mmHg) (I)**	0.0651*	0.125*	0.062*	0.228*	0.129*	0.213*	−0.182*	0.198*	0.245*	1	0.514*	0.694*	−0.023*	0.022*	−0.131*	−0.095*	−0.134*	−0.084*
	**Dia BP (mmHg) (J)**	0.1116*	0.119*	0.037*	0.245*	0.150*	0.292*	−0.186*	0.272*	0.273*	0.729*	1	0.826*	0.016	−0.022*	−0.223*	−0.154*	−0.223*	−0.157*
	**MAP# (mmHg) (K)**	0.096*	0.126*	0.045*	0.252*	0.148*	0.282*	−0.203*	0.261*	0.280*	0.880*	0.961*	1	−0.000	−0.005	−0.201*	−0.141*	−0.203*	−0.138*
	**BPM# (L)**	0.065*	0.208*	0.209*	0.064*	0.059*	0.009	−0.023	0.015	0.073*	−0.019	0.040*	0.012	1	−0.926*	−0.330*	−0.245*	−0.207*	−0.408*
	**IBI (ms) (M)**	−0.081*	−0.217*	−0.211*	−0.075*	−0.063*	−0.027	0.026	−0.032*	−0.084*	0.018	−0.045*	−0.018	−0.988*	1	0.358*	0.267*	0.2358*	0.436*
	**RMSSD# (ms) (N)**	−0.216*	−0.189*	−0.135*	−0.283*	−0.229*	−0.275*	0.061*	−0.287*	−0.237*	−0.212*	−0.329*	−0.300*	−0.504*	0.534*	1	0.585*	0.811*	0.664*
	**LF# (ms^2^) (O)**	−0.120*	−0.219*	−0.156*	−0.276*	−0.241*	−0.190*	−0.031	−0.241*	−0.190*	−0.191*	−0.235*	−0.222*	−0.347*	0.367*	0.780*	1	0.526*	0.688*
	**HF# (ms^2^) (P)**	−0.206*	−0.145*	−0.096*	−0.279*	−0.224*	−0.277*	0.055*	−0.290*	−0.228*	−0.218*	−0.327*	−0.303*	−0.329*	0.363*	0.953*	0.737*	1	0.561*
	**SDNN# (ms) (Q)**	−0.187*	−0.226*	−0.191*	−0.249*	−0.206*	−0.186*	0.016	−0.218*	−0.202*	−0.158*	−0.243*	−0.216*	−0.576*	0.597*	0.859*	0.881*	0.772*	1

# log transformed.

* = Bonferroni adjusted 2 sided significance level of α ≤ 0.05/18 = p≤0.003.

CRP = C-reactive protein. WBC = White blood count. FPG = Fasting plasma glucose. HbA_1c_ = glycosylated hemoglobin. LDL = Low density lipoprotein. HDL = High density lipoproteins. CHOL = Cholesterol. TRIG = Triglyceride. Sys BP = Systolic blood pressure. Dia BP = Diastolic BP. MAP = Mean arterial pressure. IBI = Inter-beat interval. RMSSD = Square root of the mean of successive differences. LF = Low frequency power. HF = High frequency power. SDNN = Standard deviation of normal-to-normal intervals.

**Fig 1:**
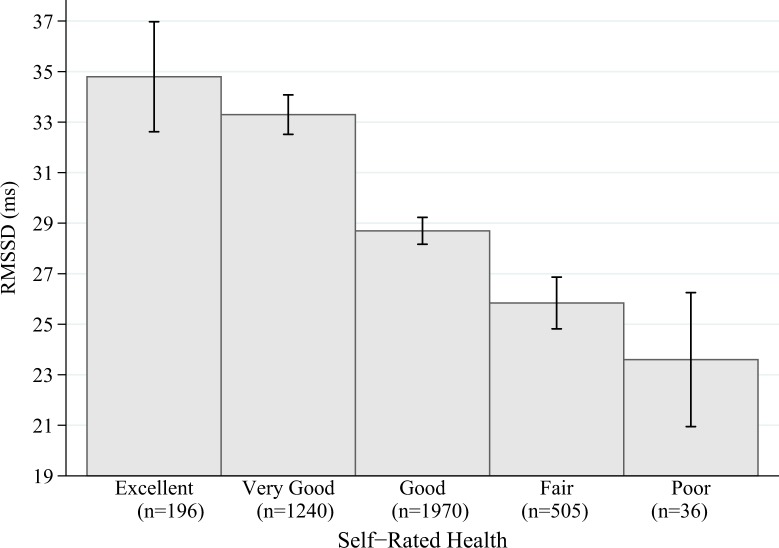
Mean RMSSD and self-rated health (error bars 95% CI).

Fig. 1 shows the mean RMSSD per SRH category and indicates a linear trend. Other biomarkers and SRH were also significantly (Bonferroni corrected) correlated but with smaller effect sizes such as blood glucose levels (FPG r = 0.13 and HbA_1c_ r = 0.13) or inflammatory markers (CRP r = 0.13 and WBC r = 0.07). It is important to note that correlations of SRH and ANS function measurements were significantly stronger (compared using Olkins *Z*) during nighttime recordings (RMSSD r = −0.21; HF r = −0.21) compared to daytime recordings (RMSSD r = −0.19; HF r = −0.19) of HRV (data not shown). Every correlation of SRH with ANS function measures was significantly larger when compared with every correlation between SRH and other biomarkers using Olkins *Z* (Table 4).

**Table 4:** Correlation strength comparison (Olkins *Z*) of self-rated health (SRH) with ANS measure or other biomarker.

	RMSSD# (ms)		SDNN# (ms)		HF# (ms^2^)		LF# (ms^2^)		BPM#	
	z-value	p-value	z-value	p-value	z-value	p-value	z-value	p-value	z-value	p-value
**FPG (mg/dl)**	**−13.7**	<0.001	**−12.68**	<0.001	**−13.29**	<0.001	**−13.07**	<0.001	−3.06	0.002
**HbA_1c_ (%)**	**−13.96**	<0.001	**−12.87**	<0.001	**−13.55**	<0.001	**−13.23**	<0.001	−3.03	0.003
**CRP# (mg/L)**	**−14.13**	<0.001	**−12.71**	<0.001	**−13.95**	<0.001	**−13.29**	<0.001	−3.22	0.001
**WBC (μL)**	**−11.86**	<0.001	**−10.34**	<0.001	**−11.6**	<0.001	**−11.05**	<0.001	−0.07	0.941
**LDL (mg/dl)**	**−13.10**	<0.001	**−12.34**	<0.001	**−12.66**	<0.001	**−12.87**	<0.001	−2.24	0.025
**HDL# (mg/dl)**	**−7.55**	<0.001	**−6.02**	<0.001	**−7.03**	<0.001	**−6.47**	<0.001	**5.20**	<0.001
**CHOL (mg/dl)**	**−12.76**	<0.001	**−11.89**	<0.001	**−12.32**	<0.001	**−12.32**	<0.001	−1.93	0.054
**TRIG (mg/dl)**	**−13.00**	<0.001	**−11.96**	<0.001	**−12.61**	<0.001	**−12.57**	<0.001	−1.97	0.048
**Sys BP (mmHg)**	**−11.43**	<0.001	**−10.44**	<0.001	**−10.97**	<0.001	**−10.85**	<0.001	−0.02	0.985
**Dia BP (mmHg)**	**−12.72**	<0.001	**−11.93**	<0.001	**−12.31**	<0.001	**−12.51**	<0.001	−2.14	0.033
**MAP# (mmHg)**	**−12.24**	<0.001	**−11.43**	<0.001	**−11.80**	<0.001	**−11.95**	<0.001	−1.40	0.162

#log transformed. Bonferroni adjusted z-value for 2 sided testing (α ≤ 0.05/54): ≥3.50 to reach statistically significance (Bold z-values).

Reading example: The null hypothesis that the correlation strength of RMSSD with SRH is equal to the correlation strength of CRP with SRH can be rejected with p<0.001. A negative z-value indicates a stronger association of ANS measure with SRH compared to the other biomarker.

CRP = C-reactive protein. WBC = White blood count. FPG = Fasting plasma glucose. HbA1c = glycosylated hemoglobin. LDL = Low density lipoprotein. HDL = High density lipoproteins. CHOL = Cholesterol. TRIG = Triglyceride. Sys BP = Systolic blood pressure. Dia BP = Diastolic BP. MAP = Mean arterial pressure. IBI = Inter-beat interval. RMSSD = Square root of the mean of successive differences. LF = Low frequency power. HF = High frequency power. SDNN = Standard deviation of normal-to-normal intervals.

**Fig 2:**
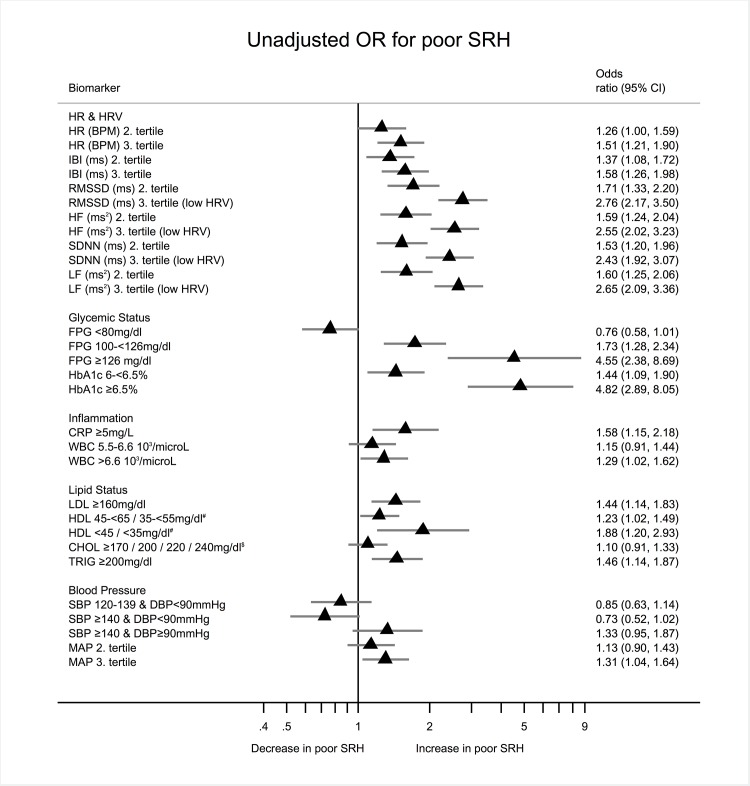
Forest plot showing unadjusted Odds Ratios (OR) of different biomarkers predicting poor self rated health: # Females / Males. $ Age <20 / 20–<30 / 30–<40 / >40. Reading example: All else being equal the odds of indicating poor SRH for participants with HbA_1c_ being larger 6.5% compared to participants with HbA_1c_ below 6% is increased by the factor 6.14.

Fig. 2 presents odds ratios and their respective 95% confidence intervals from bivariate (unadjusted) logistic regression models with poor SRH as the dependent and each biomarker as the independent variable. Clinical cut offs or highest tertiles (representing worse health) are consistently associated with higher odds ratios for poor SRH. In these bivariate analyses, HRV measures seem to have the strongest associations with SRH independent of the measure of association used.

SRH is significantly correlated (Bonferroni–corrected bivariate Pearson’s r and Kendall τ_b_) with sleep quality, work stress, age, and physical activity. Also work stress is significantly and positively correlated with all measures of blood glucose, blood lipids (not HDL), diastolic BP, as well as negatively correlated with all measures of ANS function. All correlations had a small effect size (Table 5). Socioeconomic status measures, gender, and smoking status were not associated with poor SRH (not bivariate nor in multivariate models) and thus were excluded from subsequent models.

**Table 5:** Bonferroni corrected Pearson’s correlation and Kendall τ_b_ for potential confounders with self-rated health (SRH).

		Kendall τ_b_							
	Covariates	SRH	(A)	(B)	(C)	(D)	(E)	(F)	(G)
Pearson’s Correlation	**SRH**	1	0.200*	0.154*	0.043*	0.303*	0.217*	0.023	−0.011
	**Age (decade) (A)**	0.252*	1	0.024*	0.135*	0.107*	0.136*	−0.218*	0.139*
	**Sport (B)**	0.177*	0.036*	1	−0.025	0.056*	0.046*	−0.006	0.028*
	**Alcohol (C)**	0.056*	0.146*	0.005	1	0.071*	0.041*	−0.039*	0.043*
	**Sleep Quality (Jenkins) (D)**	0.397*	0.161*	0.073*	0.082*	1	0.158*	0.028*	−0.020
	**Work Stress (ERI) (E)**	0.271*	0.191*	0.036*	0.042*	0.240*	1	−0.096*	0.084*
	**Hierarchy (F)**	0.028	−0.288*	−0.005	−0.000	0.055*	−0.090*	1	−0.581*
	**Span of Control (G)**	−0.008	0.174*	0.032*	0.029	−0.020	0.073*	−0.639*	1

* = Bonferroni adjusted 2 sided significance level of α ≤ 0.05/8 = p≤0.006.

**Alcohol** = No alcohol, 1–2 times/month, 1–2 times/week, 3–5 days/week, 6–7 days/week. **Sport** = Sweat rich activities 3 or more times/week, 2 times/week, 1/week, 1–3 times/month, less than 1/month.

### Multivariate associations

**Fig 3:**
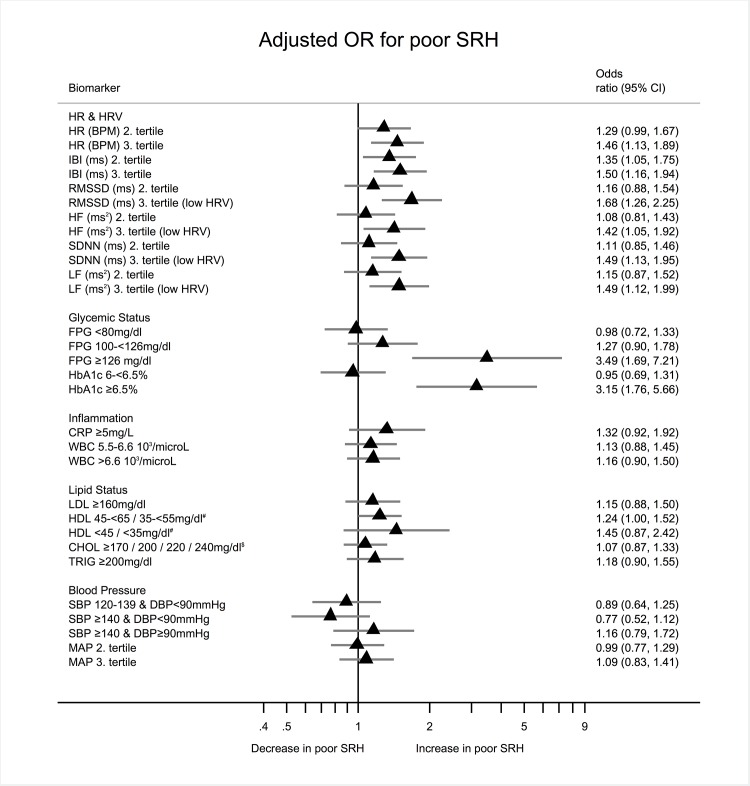
Forest plot showing adjusted Odds Ratios (OR) of different biomarkers predicting poor self rated health; # Females / Males. $ Age <20 / 20–<30 / 30–<40 / >40. Adjusted for age, sport, alcohol, sleep quality, and work stress. Reading example: All else being equal the odds of indicating poor SRH for participants with HbA_1c_ being larger 6.5% compared to participants with HbA_1c_ below 6% is increased by the factor 3.13.

The forest plot (Fig. 3) presents the same models but adjusted for age, lifestyle, and work stress. Adjusted logistic regression models showed a negative association with measures of HRV comparing the third (worse health) vs. first (better health) tertile of vagally mediated HRV (RMSSD OR = 1.64 95%CI [1.23–2.19] *p = 0.001*; HF OR = 1.38 [1.03–1.85] *p = 0.034*) and both sympathetic and parasympathetic mediated HRV (SDNN OR = 1.46 [1.12–1.91] *p = 0.006*; LF OR = 1.39 [1.05–1.84] *p = 0.021*). Glycemic markers (HbA_1c_ >6.5% OR = 2.90 [1.65–5.09] *p<0.001*; FPG >126mg/dl OR = 3.49 [1.72–7.06] *p = 0.001*) were positively associated with poor SRH. No adjusted association was found with any inflammatory markers, BP, and blood lipid measures (Fig. 3). Work stress and sleep quality were significantly, and independent of HRV, positively associated with poor SRH in all models (ERI OR 2.3 [1.89–2.80] *p <0.001*; Jenkins OR 1.2 [1.18–1.24] *p<0.001*). This means, the higher the self-reported work-stress the higher the odds ratio for worse SRH. The OR of the HRV variables were slightly attenuated after entering work stress and sleep quality into the models but remained significant and of a relevant effect size. Full models had a pseudo–R^2^ between 17–19%.

## Discussion

### Principal findings

This is the first study to show that a global measure of SRH was associated with ANS function as indexed by HRV. The strength of associations of traditional biomarkers with ANS function measures were compared and it was shown that all measures of ANS function were significantly more strongly associated with SRH than any of the other assessed biomarkers in a healthy working population. These findings provide valuable information about the mechanisms by which SRH is related to mortality and morbidity.

### Comparison with existing studies

Consistent with prior research, the present study found that biomarkers associated with inflammation (CRP and WBC) and the MetS (FPG and HbA_1c_) had significant bivariate associations with SRH. The magnitudes of the associations in the present study are similar to those previously reported [17,22,24,36] but somewhat smaller than reported by others [18]. For example, our unadjusted association between SRH and CRP of r = −0.11 is similar to the −0.16 association found by Janszky [36] but smaller than the r = −0.28 association found by Christian [18]. The reasons for this difference are unclear. Both the Janszky [36] and Christian [18] samples were older than the present sample and one [36] sample was only women whereas our sample was significantly larger and primarily men (Total N = 3357 versus N = 232 for [36] and N = 250 for [18]. However our results are consistent with the majority of the extant literature whereas the Christian [18] findings are somewhat larger. Importantly in the adjusted models, the differences between our findings (r = −0.07) and the Christian [18] study (r = −0.12) narrowed and remained consistent with the Janszky [36] study (r = −0.12). Furthermore, the reported associations between HRV measures and the other biomarkers are also comparable to the existing literature e.g. [24–26,29,37,38]. Thus the present findings with respect to the association between inflammatory and MetS biomarkers, and SRH are comparable to others and support the generalizability of the present findings.

Consistent with our hypothesis HRV is a more inclusive and integrative index than other frequently investigated biomarkers. There are several compelling reasons why this might be the case. First, it has been shown consistently that measures of vagally-mediated HRV have strong independent associations with mortality and morbidity [29,39] and so does SRH [1]. Second, measures of vagally-mediated HRV are independently associated with a range of biomarkers including inflammatory and MetS biomarkers [24–26]. Third, vagally-mediated HRV is associated with emotional regulation and dysregulation [40,41]. The latter two points show that vagally-mediated HRV is associated with both biological measures as well as psychological measures and thus might be more inclusive just as SRH is.

### Possible explanations and implication

The vagus nerve innervates a wide range of organ systems in the body and as such is well positioned to provide feedback about the state of the organism to the brain. In fact 80% of the vagal fibers are afferent and thus the vagus has been shown to be important in transmitting diverse types of information to the brain including information concerning immune status, blood glucose levels, and pain [42–44]. It is now widely accepted that the ANS is also important in immune function [45,46]. For example, the vagus nerve is known to relay information about peripheral immune status to the brain via interleukin-1 cytokine receptors conveyed by paraganglia cells situated in parasympathetic ganglia [47]. In addition, efferent vagal activity via the release of acetylcholine inhibits release of pro-inflammatory cytokines and has been termed the cholinergic anti-inflammatory pathway [48].

It is less well known that the vagus is also involved in glucose regulation. Vagal afferents in the hepatic portal contain glucagon-like peptide-1 receptors that convey information about peripheral glucose status to the brain. In addition, vagal efferent fibers innervate the liver and the kidneys and play a role in glucose regulation [49–51]. The association between vagal activity and glucose levels has been previously shown to be stronger during nighttime [24]. Overall, the action of the vagus nerve is a common denominator for both immune and glucose regulation. Moreover the vagus nerve is also important in pain regulation [44,52–54].

Thus measures of HRV may capture a broader range of function across various bodily systems than other biomarkers. Taken together the literature suggests an important role for the ANS, particularly the vagus nerve, in the transmission of important information about bodily functions to the brain. Importantly, efferent vagal activity also seems to be significant in regulating these same bodily functions. This role is also supported by our result of a significantly stronger correlation between SRH and vagally mediated HRV during nighttime (as opposed to daytime values) a period of rest where the parasympathetic nervous system and hence the vagus is expected to be dominant. Better sleep quality is associated with better SRH [55] and insufficient sleep is associated with poor SRH [56,57]. Likewise, better sleep quality is associated with higher measures of heart rate variability [58]. Additional analysis from logistic regression models on poor SRH adjusted for age, sex, sport, and alcohol reveal higher OR for poor SRH with lower tertiles of HRV in the nighttime models (compared to daytime models). Coefficients are attenuated after additional adjustment for sleep quality, but the patterns and statistical significances remain. This leads to the conclusion that sleep quality accounts for some but not all differences between day and nighttime measures of HRV predicting SRH. Thus, HRV may serve as an important index of this bi-directional communication across diverse bodily systems.

The exact neural concomitants of these bodily functions have also been a topic of investigation. A network of neural structures has been identified that is important in the integration and regulation of many systems of the body and has been termed the medial prefrontal-brainstem axis [59]. An important component of this network is the prefrontal cortex (PFC) that has been linked with immune and glucose regulation. For example, Page et al. [60] have reported that higher circulating glucose levels were associated with greater activity in the medial PFC. Activity in this area increased in response to glucose infusion and was related to decreased interest in food stimuli. Importantly, this inhibitory control over food motivation was absent in obese individuals. In addition, it was recently shown that activity in a network including the medial PFC was reduced in response to glucose ingestion [61]. These findings suggest that the PFC is an important site for the regulation of glucose and eating behavior such that greater activity in this region is associated with euglycemia and context appropriate eating behavior. Of particular relevance to the present study, we have reported that HRV is directly related to activity in the medial PFC [23].

### Model of neurovisceral integration

A possible explanation for the stronger negative association of HRV and SRH can be drawn from the neurovisceral integration model of cardiac vagal control. This model integrates autonomic, attentional, and affective systems into a functional and structural network [62]. One of the major structures of this network, the ventromedial PFC is particularly involved in building the meaning of a situation, especially when conceptual information drives affective, physiological, and behavioral responses [63]. Furthermore, frontal cortex structures are particularly involved in visceral sensory processing with an emphasis on the medial prefrontal region [64]. Although SRH is likely to be associated with a distributed brain system rather than a single brain center, the ventromedial PFC could be the nexus between SRH and HRV where the complete information is integrated and processed either consciously or more likely non-consciously.

Additionally, the bi-directional communication between the peripheral nervous system and the brain has been clearly enunciated in the neurovisceral integration model [65–68]. The current study therefore supports the hypothesis that the extent of central –peripheral neural feedback and central autonomic network– ANS integration, as indexed by HRV [62,69] is associated with SRH.

Four possible factors have been proposed to explain the validity of SRH as a predictor of health outcomes [9]. Importantly, HRV appears to share many of these factors. First, both SRH and HRV are more inclusive than other biomarkers or disease reports and may be based at least in part on bodily sensations that are accessible only to the individual [11]. Second, they may reflect changes over time in health status [70]. Third, both SRH and HRV may influence (health) behavior. This might be a function of the ability to read, interpret, and react to information from within the body (interoception), the outer world (exteroception) and its specific context, and therefore may guide health-related behavior such as eating. Fourth, both SRH and HRV reflect resources, which enable persons to cope with health threats.

### Strengths and limitations of this study

This study adds to the ample evidence of the validity of a single SRH item as a measure of one’s health. However, a single item may have also its limits, which should be further explored. For example, research has identified certain subpopulations such as women or Blacks where the SRH–mortality relationship is less pronounced. Mastery and a more active lifestyle in a variety of domains such as cognitive, physical and social domains has been proposed as a potential explanation in this regards [71]. There are also some other limitations to this study: The relatively weak associations of biomarkers with SRH might be due to low numbers of observations in the categories “fair” and “poor health”, which is not an unusual observation in an occupational cohort due to a potential “healthy worker bias”. However percentages are comparable to others found in the literature e.g. [72,73]. Due to the cross-sectional design of our study causal directions or temporal associations between SRH and biomarkers cannot be concluded. However the predictive value of SRH on mortality or morbidity or the sensitivity to change have been reported elsewhere [70].

This study has several strengths. This is a large occupational sample from four distinct geographical regions within Germany comprised of employees with differing socioeconomic backgrounds rather than being limited to one particular professional group. Furthermore, all participants were from a similar cultural background, so genetic and cultural heterogeneity that might affect SRH are likely minimized. Finally this study accounts for many potential covariates in the statistical analyses and HRV was recorded over 24h.

## Conclusion

In sum we report for the first time that poor SRH was inversely associated with vagally-mediated HRV in a large, young healthy working cohort. It has been shown that HRV was more strongly associated with SRH than any other assessed biomarker. The present study provides important insights into the mechanisms by which SRH is able to provide information not readily available from other sources about the health status of individuals. The common ability of both SRH and HRV to provide an integrative index of exteroceptive and interoceptive information relevant to health related perceptions and behaviors offers new insights into SRH and HRV. Future research is needed to investigate the temporal and causal association of ANS function and self-rated health.
